# P53/PANK1/miR‐107 signalling pathway spans the gap between metabolic reprogramming and insulin resistance induced by high‐fat diet

**DOI:** 10.1111/jcmm.15053

**Published:** 2020-02-12

**Authors:** Lu Yang, Bin Zhang, Xinju Wang, Zhenhua Liu, Juan Li, Shumiao Zhang, Xiaoming Gu, Min Jia, Haitao Guo, Na Feng, Rong Fan, Manjiang Xie, Jianming Pei, Li Chen

**Affiliations:** ^1^ Department of Physiology National Key Discipline of Cell Biology Fourth Military Medical University Xi'an China; ^2^ Department of Aerospace Physiology Fourth Military Medical University Xi'an China; ^3^ Battalion 5 of Cadets Fourth Military Medical University Xi'an China

**Keywords:** high‐fat diet, insulin resistance, metabolic reprogramming, MiR‐107, P53, PANK1

## Abstract

High‐fat diet (HFD) leads to obesity, type II diabetes mellitus (T2DM) and increases the coincidence of cardiovascular diseases and cancer. Insulin resistance (IR) is considered as the ‘common soil’ of those diseases. Furthermore, people on HFD showed restrained glycolysis and enhanced fatty acid oxidation, which is the so‐called metabolic reprogramming. However, the relationship between metabolic reprogramming and IR induced by HFD is still unclear. Here, we demonstrate that PANK1 and miR‐107 were up‐regulated in the liver tissue of mice on HFD for 16 weeks and involved in metabolic reprogramming induced by palmitate acid (PA) incubation. Importantly, miR‐107 within an intron of PANK1 gene facilitated IR by targeting caveolin‐1 in AML12 cells upon PA incubation. Moreover, we identify that HFD enhanced P53 expression, and activation of P53 with nutlin‐3a induced PANK1 and miR‐107 expression simultaneously in transcriptional level, leading to metabolic reprogramming and IR, respectively. Consistently, inhibition of P53 with pifithrin‐α hydrobromide ameliorated PA‐induced metabolic reprogramming and IR. Thus, our results revealing a new mechanism by which P53 regulate metabolism. In addition, the results distinguished the different roles of PANK1 and its intron miR‐107 in metabolic regulation, which will provide more accurate intervention targets for the treatment of metabolic diseases.

## 1 INTRODUCTION

Long‐term high‐fat diet (HFD) will cause severe insulin resistance (IR) which is central to the pathophysiology of the metabolic syndrome and type II diabetes mellitus (T2DM). The mechanism by which HFD results in IR includes excess lipid accumulation, increased reactive oxygen species (ROS) and production of inflammatory cytokines by macrophages.^1^ However, the scenario of IR induced by HFD has not been fully depicted.

Liver is the centre of metabolism, and IR in liver is enough to evoke hyperglycaemia and glucose intolerance. In the liver, IR is manifested by the blunted ability of insulin to suppress hepatic glucose production.^2^ Mice with hepatocytes lacking insulin receptors showed severe glucose intolerance and impaired ability of insulin to inhibit liver glucose output.^3^ During fasting, glycolysis is inhibited but fatty acid oxidation (FAO) and gluconeogenesis in the liver are increased, which is critical to maintain the glucostasis. This transition of metabolic substrate utilization termed as metabolic reprogramming, which is induced by many factors such as hypoxia, nutrition deprivation and tumour.^4^, ^5^ However, the relationship between metabolic reprogramming and IR induced by HFD is not fully elucidated.

The coenzyme A (CoA) was recently identified as a mediator of the gluconeogenesis and FAO.^6^ Pantothenate kinase (PANK) catalyses the first committed step and controls the overall rate of CoA biosynthesis.^6^ In mammals, there are three genes that encode four characterized isoforms of PANK. PANK1α and PANK1β are from the same gene and arise from alternate initiation exons, whereas the PANK2 and PANK3 isoforms are encoded by distinct genes. PANK1 is required for the hepatic CoA increase during the transition from glucose utilization to FAO that occurs in the fasted state.^6^ PANK1 is highly expressed in the liver and corresponds to the liver possessing the highest concentration of CoA.^7^ Deletion of PANK1 in *ob/ob* mice dramatically suppressed hepatic gluconeogenesis, hyperglycaemia and hyperinsulinemia without affecting insulin signalling.^4^ However, the role of PANK1 in metabolic reprogramming and IR induced by HFD is unclear.

MiR‐103 and miR‐107 are paralogs, which differ only at a single nucleotide near the 3’ end of the miRNAs. For all known vertebrate species, each miR‐103/107 paralog exists within an intron in the gene, which encodes the PANK. Overexpression of miR‐103 or miR‐107 in mice induced IR indicated by glucose and insulin intolerance and impaired insulin signalling pathway.^8^ Expression levels of intronic miRNAs and their host genes are often highly correlated, presumably because they are co‐transcribed.^9^ Considering PANK1 affects gluconeogenesis and FAO, the miR‐107 and their ‘host’ gene *PANK1* may act synergistically to regulate glucose/lipid metabolic transition and contribute to the IR induced by HFD.

The previous study confirmed that PANK1 locus was activated by P53 and the coregulation of PANK1 and its intronic miRNA‐107 were characterized.^10^ As inhibition of P53 attenuates steatosis and liver injury in a mouse model of non‐alcoholic fatty liver disease induced by HFD,^11^ we assume that HFD may promote the co‐transcriptional activation of PANK1 and miRNA‐107 by activating P53, which leads to the metabolic reprogramming and IR.

In present study, we found that HFD induced metabolic reprogramming and IR through P53 transcriptional activation of PANK1 and miR‐107, respectively. Our results provide a new mechanism that P53 regulates metabolism and distinguish different effects of host gene and its intron miRNA on metabolism regulation.

## 2 MATERIALS AND METHODS

### 2.1 Animals and treatment

Thirty‐six six‐week‐old male C57BL/6 mice (17‐20 g) were provided by the animal centre of Fourth Military Medical University. They were housed in separated cages (20°C‐22°C, fed ad libitum, and maintained on a 12‐hour light/12‐hour dark cycle). At the age of 8 week, mice were numbered according to random number table, ranked by the ascending order and randomized into six groups: standard normal diet (CON) and high‐fat diet (HFD) and maintained on a standard normal diet or HFD (60% fat, Research Diet, USA) for 1, 2, 4, 8 and 16 weeks.^12^ Bodyweights were recorded every week. After completing the feeding regimen, the animals were fasted for 12 hours before anaesthetized with pentobarbital sodium (50 mg/kg i.p.) and livers were collected for further analyses. All procedures involving animals were performed according to the National Institutes of Health Guidelines on the Use of Laboratory Animals (NIH Publications No. 8023, revised 1978) and were approved by the Fourth Military Medical University Committee on Animal Care.

### 2.2 Intraperitoneal glucose and insulin tolerance tests

Six mice from each group were measured. For the intraperitoneal glucose tolerance test (IPGTT), after a 12‐hour period of fasting, 2 mg/g bodyweight glucose was administered via intraperitoneal injection to age‐matched control and HFD mice. Blood samples were obtained from the tail vein at 0, 15, 30, 60, 90 and 120 minutes after injection and measured using a glucometer (Omron, Kyoto, Japan). For the intraperitoneal insulin tolerance test (IPITT), after a 6‐hour period of fasting, insulin (0.75 U/kg) was injected. Blood samples were obtained from the tail vein at 0, 15, 30, 60, 90 and 120 minutes after injection and measured with a glucometer.

### 2.3 Quantitative real‐time RT‐PCR

The real‐time PCR experiment was performed according to the MIQE guidelines.^13^ Total RNA including miRNAs was extracted from flash‐frozen tissue or cells using RNAiso (Takara, Japan), and cDNA was synthesized from RNA via Mir‐X miRNA First‐Strand Synthesis Kit (TaKaRa, Japan). Expression analysis of the reported genes was performed by real‐time PCR with SYBR Premix Ex TaqTM (TaKaRa, Japan). Data were analysed via the relative Ct (2^−ΔΔCt^) method and were expressed as a fold change compared with the respective control (U6 or β‐actin). Primers were designed and synthesized by Sangon corporation (Shanghai, China), and the sequences were listed in Table S1.

### 2.4 Protein extraction and Western blotting

Tissue samples were grinded a glass homogenizer in T‐PER Tissue Protein Extraction Reagent (Thermo, USA) with 1% protease and phosphatase inhibitor cocktail (Thermo). Protein concentration was determined by bicinchoninic acid (BCA) Protein Assay Kit (Thermo). Equivalent amount of proteins were separated on a 4%‐12% Bis‐Tris gels (Thermo), transferred to PVDF membrane (Millipore) and incubated overnight at 4°C with antibodies directed against PANK1 (1:1000, Proteintech Group), PANK2 (1:1000, Abcam), PANK3 (1:1000, Novus), P53 (1:1000, CST), Phosphoinositide 3‐kinase (PI3K, 1:1,000, CST), Akt (1:1,000, CST), phosphor (p)‐Akt (Ser 473) (1:1000, CST), fatty acid synthase (FASN, 1:1000, CST), stearoyl‐CoA desaturase 1 (SCD1, 1:1000, CST), carnitine palmitoyltransferase 1α (Cpt1α, 1:1000, Abcam), insulin receptor β (IRβ, 1:1000, Abcam), Caveolin‐1 (Cav1, 1:1000, Abcam) or β‐actin (1:1000, Proteintech Group). After washing, blots were incubated for 1h with horseradish peroxidase (HRP)‐conjugated goat anti‐rabbit IgG (1:10 000) or goat antimouse IgG (1:10 000) at room temperature. The blots were developed with a chemiluminescent HRP substrate (Millipore). The immunoblot was visualized with ChemiDocXRS (Bio‐Rad), and the blot densities were analysed with Quantity One software.

### 2.5 Haematoxylin‐eosin staining

Liver tissue was fixed in 10% formalin for paraffin. Paraffin‐embedded liver tissue was used for Haematoxylin‐eosin (H&E) staining as described.^14^

### 2.6 Cell culture

AML12 cells were obtained from Type Culture Collection of the Chinese Academy of Sciences and cultured in DMEM/F‐12 supplemented with 10% foetal bovine serum (FBS) (Gibco), 1% ITS (Gibco), 40 ng/mL Dexamethasone (Sigma‐Aldrich) and incubated in a humidified incubator at 37°C and 5% CO_2_. AML12 cells were cultured in the medium without the supplementation of insulin at the beginning of all treatment. The cells at 70% confluence were exposed to palmitate acid (Sigma‐Aldrich), nutlin‐3a or pifithrin‐α hydrobromide (Med Chem Express, USA) for 24 hours. For the insulin stimulation experiments, the cells were treated with or without 100 nmol/L insulin for 20 minutes.

### 2.7 Plasmid and oligonucleotides transfection

The DNA plasmid GV141‐PANK1 and candidates small interfering RNA (siRNA) targeting PANK1 mRNA and their scrambled RNA were purchased from Genechem. The mimics or inhibitors for miRNA were purchased from Ribobio. Transfection into AML12 cells was performed with Lipofectamine 3000 (Invitrogen) according to the protocol. The transfected cells were cultured for 48 hours and used for further experiments.

### 2.8 Assays of G6Pase, GCK, PFK‐1 and PEPCK activity

The activity of glucose 6 phosphatase (G6Pase), phosphoenolpyruvate carboxykinase (PEPCK), glycolytic enzymes glucokinase (GCK) and phosphofructokinase‐1 (PFK‐1) was determined with the G6Pase assay kits, PEPCK (Solarbio Life Sciences), hexokinase activity assay kit (Abcam) or phosphofructokinase activity colorimetric assay kit (Sigma‐Aldrich) following the manufacturer's instructions.

### 2.9 Glucose production assay

The glucose production of AML12 cells was detected as described previously.^15^ Briefly, after treatment cells were washed and incubated for 2 hours in glucose‐, L‐glutamine‐, phenol red‐, sodium pyruvate‐ and sodium bicarbonate‐free DMEM supplemented with or without gluconeogenic substrates (200 μmol/L glycerol only or 10 mmol/L sodium lactate and 2 mmol/L sodium pyruvate). Glucose in the medium was measured using a colorimetric glucose assay kit (Cat: K606‐100, BioVison) and normalized with total cellular protein.

### 2.10 Oil red O staining

Frozen liver tissue sections were stained with oil red O for lipid detection as described.^16^ The lipid content in cultured cells was determined by oil red O staining (Abcam). Briefly, the cells were washed with phosphate buffer solution. Cells were fixed by propylene glycol for 2 minutes and incubated in oil red O Solution for 6 minutes and then differentiate the cells in 85% propylene glycol for 1 minute. Wash the 6‐well plates with distilled water and incubate the cells with haematoxylin for 1 minute and rinse thoroughly. Images of plates of oil red O were acquired using an inverted Eclipse TE300 microscope (Nikon).

### 2.11 Statistical analysis

All statistical analyses were performed using Prism 8.0 (GraphPad Software), and all data are expressed as the mean ± SEM. Statistical differences were assessed by one‐way ANOVA (followed by the post hoc Tukey‐Kramer test). A value of *P* < .05 was considered statistically significant.

## 3 RESULTS

### 3.1 HFD induced metabolic reprogramming and IR in mice

**Figure 1:**
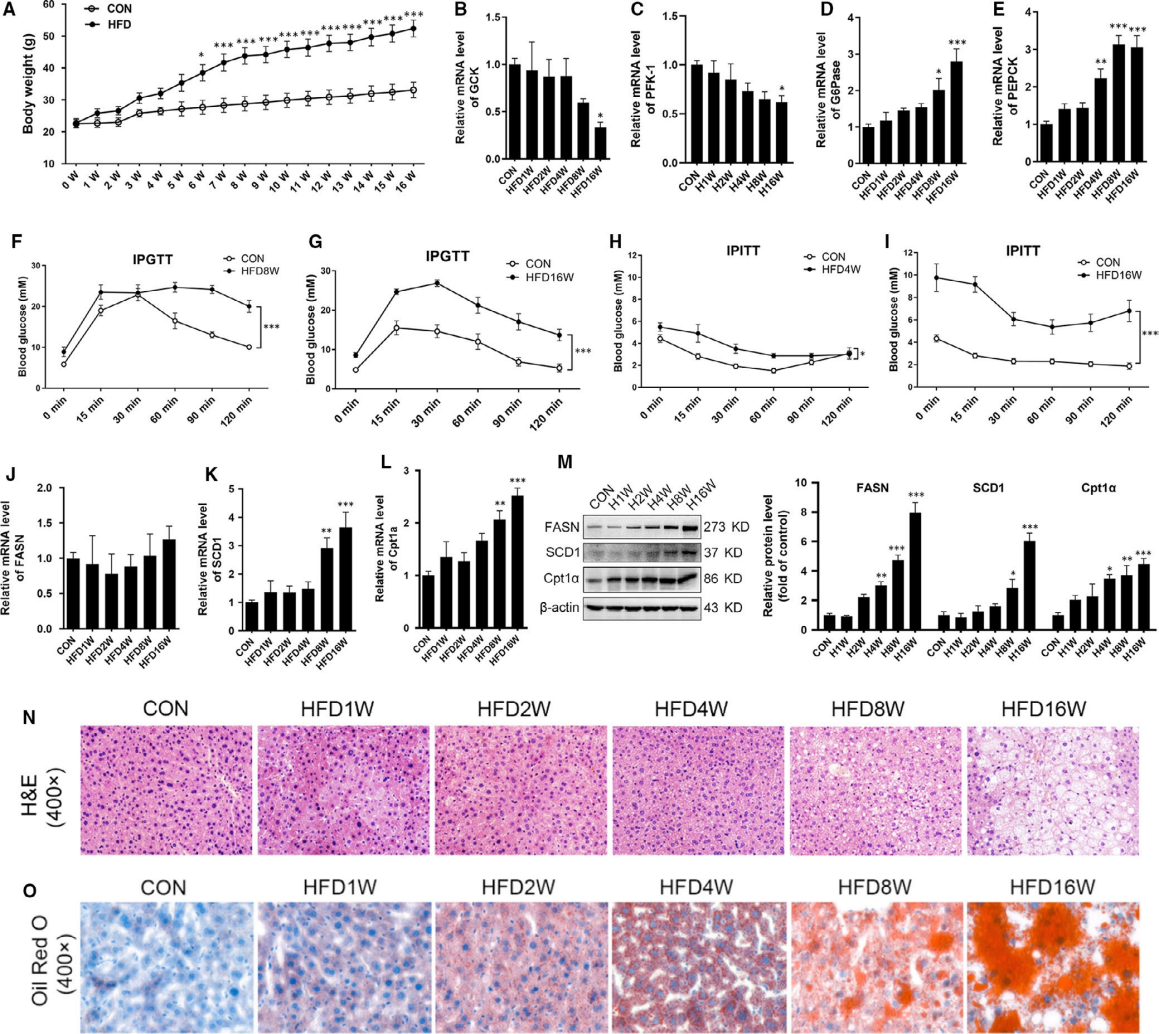
High‐fat diet induced metabolic reprogramming and insulin resistance in mice. Eight‐week‐old male C57BL/6 mice were randomized into two groups: standard normal diet (CON) and high‐fat diet (HFD). The mice maintained on a standard normal diet or HFD (60% fat) for 16 wk. A, Bodyweight was measured weekly. B‐E, The mRNA of glucokinase (GCK), phosphofructokinase‐1 (PFK‐1), glucose 6 phosphatase (G6Pase) and phosphoenolpyruvate carboxykinase (PEPCK) in the liver tissue of mice was measured. F and G, Intraperitoneal glucose tolerance test (IPGTT) was performed in mice on HFD for 8 and 16 wk. H and I, Intraperitoneal insulin tolerance test (IPITT) was performed in mice on HFD for 4 and 16 wk. J‐L, The mRNA of fatty acid synthase (FASN), stearoyl‐CoA desaturase 1 (SCD1) and carnitine palmitoyltransferase 1 (Cpt1α) in the liver tissue of mice was measured. M, The protein levels of FASN, SCD1 and Cpt1α in the liver tissue were measured. N and O, Haematoxylin‐eosin (H&E) and oil red O staining revealed markedly enhanced micro‐ and macrovesicular steatosis in the livers of mice on HFD. Representative images are shown (400×). All values are presented as mean ± SEM. *P < .05, **P < .01, ***P < .001 (vs CON). n = 6

During 16 weeks of HFD feeding, bodyweight was measured every week and HFD induced significant weight gain at the 6th week (Figure 1A). Metabolic reprogramming was an adaptation to the local environment, which was first identified in tumour and recognized as a hallmark of cancer.^17^ It was reported that HFD induced the alteration of nutrients metabolism, which is also regarded as metabolic reprogramming.^18^ Our results showed that the mRNA expressions of GCK and PFK‐1 involved in glycolysis were suppressed (Figure 1B,C) whereas G6Pase and PEPCK modulating gluconeogenesis were enhanced in the liver of mice during HFD feeding (Figure 1D,E). This alteration reflected the systemic IR which was further validated by IPGTT and IPITT which were performed at 1st, 2nd, 4th, 8th and 16th week of HFD feeding (Figure S1). IPGTT results showed that glucose tolerance impairment occurred at the 8th week of HFD (Figure 1F‐G) and IPITT results showed significant difference in HFD mice at the 4th week (Figure 1H‐I). Meanwhile, the level of lipid metabolism was determined. In the liver of mice on HFD, the mRNA and protein level of key enzymes (FASN and SCD1) involved in lipogenesis were increased, and the expression of FAO enzyme Cpt1α was up‐regulated significantly (Figure 1J‐M). Furthermore, the histopathological evaluation of formalin‐fixed, haematoxylin‐eosin‐stained liver slides revealed more micro‐ and macrovesicular steatosis in the livers of HFD‐fed mice compared with the mice on the control diet (Figure 1N). Oil red O staining quantification was performed on the flash‐frozen liver tissues and revealed that the intrahepatic ectopic lipid accumulation was increased in HFD‐fed mice compared with the mice on the control diet (Figure 1O). These findings suggested an adaptive enhancement of lipid metabolism upon HFD.

Moreover, PA incubation suppressed glycolysis of AML12 as indicated by the decreased mRNA level and enzyme activity of GCK and PFK‐1 (Figure S2A‐D). The mRNA level and enzyme activity of G6Pase and PEPCK involved in gluconeogenesis were enhanced by PA incubation in a dose‐dependent manner (Figure S2E‐H). Moreover, glucose production was enhanced by PA incubation in a dose‐dependent manner (Figure S2I). The key enzymes involved in lipid metabolism were also up‐regulated (Figure S2J). The IR induced by PA was verified by the decreased phosphorylation of Akt (Figure S2K). These data confirmed that both of HFD and PA led to metabolic reprogramming and IR simultaneously, which implied that metabolic reprogramming and IR were accompanied intimately.

### 3.2 PANK1 was up‐regulated in the liver tissue of mice on HFD and involved in metabolic reprogramming induced by PA

**Figure 2:**
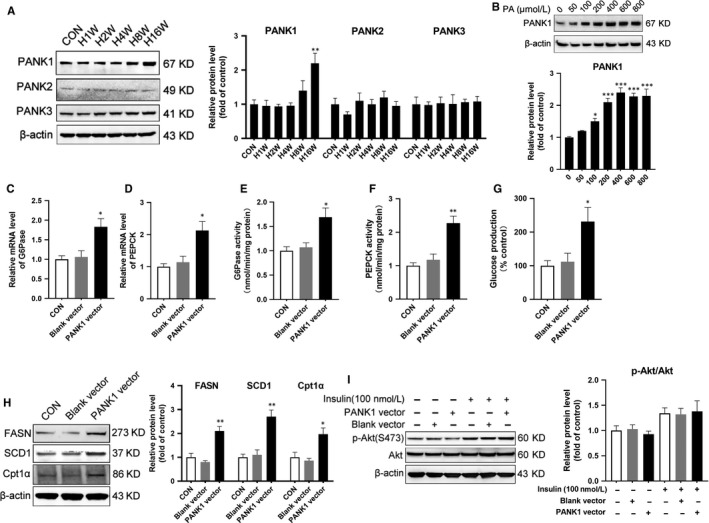
PANK1 was up‐regulated in liver tissue of mice on high‐fat diet and in cultured hepatocytes treated with palmitate acid. A, The mice were fed on HFD, and the protein levels of PANK1, PANK2 and PANK3 in the liver tissue were measured by Western blotting (n = 6). B, The AML12 cells were treated with 0, 50, 100, 200, 400, 600 and 800 μmol/L palmitate acid (PA) for 24 hours, and the protein level of PANK1 was measured by Western blotting (n = 3 independent experiments). PANK1 vector or blank vector was transfected into AML12 cells with lipofectamine. The mRNA level of G6Pase (C) and PEPCK (D) in AML12 cells were measured by real‐time RT‐PCR. The activity of G6Pase (E) and PEPCK (F) and glucose production (G) was measured in AML12 cells. The protein levels of FASN, SCD1 and Cpt1α were measured by Western blotting (H). I, Before harvest, cells were stimulated with 100 nmol/L insulin for 20 min and protein expressions of p‐Akt (S473) and Akt were measured by Western blotting. All values are presented as mean ± SEM. Data in A and B, *P < .05, **P < .01, ***P < .001 (vs CON). Data in the rest, *P < .05, **P < .01 (vs Blank vector), n = 3 independent experiments

Pank1 was required to support the metabolic transition from the fed to the fasted state through modulating the biosynthesis of CoA.^6^ The increase in hepatic CoA during fasting was blunted in the *Pank1* ^−/−^ mouse and resulted in reduced FAO.^4^ During HFD, the enhanced FAO and suppressed glycolysis were like the metabolic transition from the fed to the fasted, so we investigated the role of PANK in this metabolic transition. All of three isoforms of PANK were measured by Western blotting in the liver of mice, and only the expression of PANK1 was significantly up‐regulated induced by HFD (Figure 2A). PA incubation also significantly increased the expression of PANK1 (Figure 2B). Then, we investigated the influence of PANK1 on metabolic reprogramming. Overexpression of PANK1 enhanced the expression and activity of enzymes modulating gluconeogenesis and glucose production (Figure 2C‐G). Moreover, overexpression of PANK1 significantly increased the expression of enzymes involved in lipid metabolism (Figure 2H). However, overexpression of PANK1 had no influence on the insulin signalling (Figure 2I).

**Figure 3:**
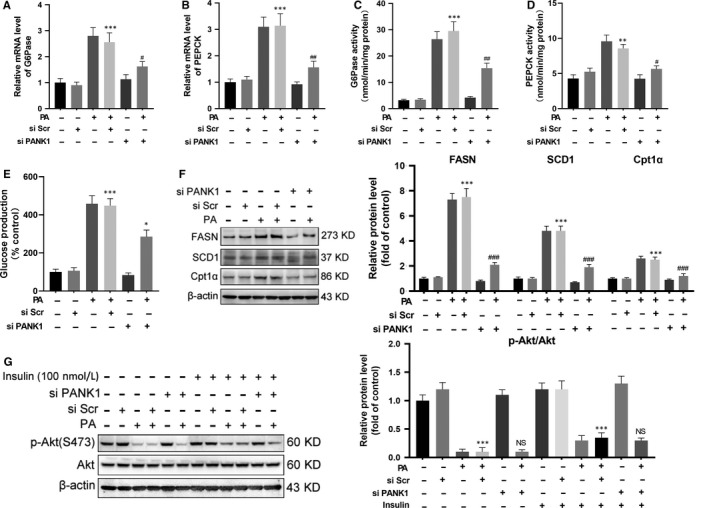
Knockdown PANK1 ameliorated metabolic reprogramming but failed to improve insulin resistance induced by palmitate acid. The AML12 cells were treated with scrambled small interfering RNA (si Scr) or PANK1 small interfering RNA (si PANK1). After 24 h, the cells were exposed to palmitate acid (600 nmol/L, PA) for 24 h. A and B, The mRNA level of G6Pase and PEPCK in AML12 cells was measured by real‐time RT‐PCR. C and D, The activity of G6Pase and PEPCK in AML12 cells was measured. E, Glucose production measured in AML12 cells. F, The protein levels of FASN, SCD1 and Cpt1α in AML12 cells were measured by Western blotting. G, Before harvest, cells were stimulated with 100 nmol/L insulin for 20 min and protein expressions of p‐Akt (S473) and Akt were measured by Western blotting. All values are presented as mean ± SEM. **P < .01, ***P < .001 (vs si Scr); ^#^ *P* < .05, ^##^ *P* < .01, ^###^ *P* < .001 (vs PA + si Scr), n = 3 independent experiments

Furthermore, we investigated whether PANK1 was indispensable in metabolic reprogramming induced by PA. Knocking down PANK1 by siRNA partially reversed the enhanced gluconeogenesis induced by PA (Figure 3A‐E). Meanwhile, si PANK1 transfection significantly abolished the up‐regulation of enzymes involved in lipid metabolism induced by PA (Figure 3F). However, knocking down PANK1 showed no influence on insulin signalling (Figure 3G). Thus, although PANK1 was indispensable in metabolic reprogramming, the insulin signalling was PANK1‐independent.

### 3.3 MiR‐107 within an intron of PANK1 gene induced IR by targeting caveolin‐1

**Figure 4:**
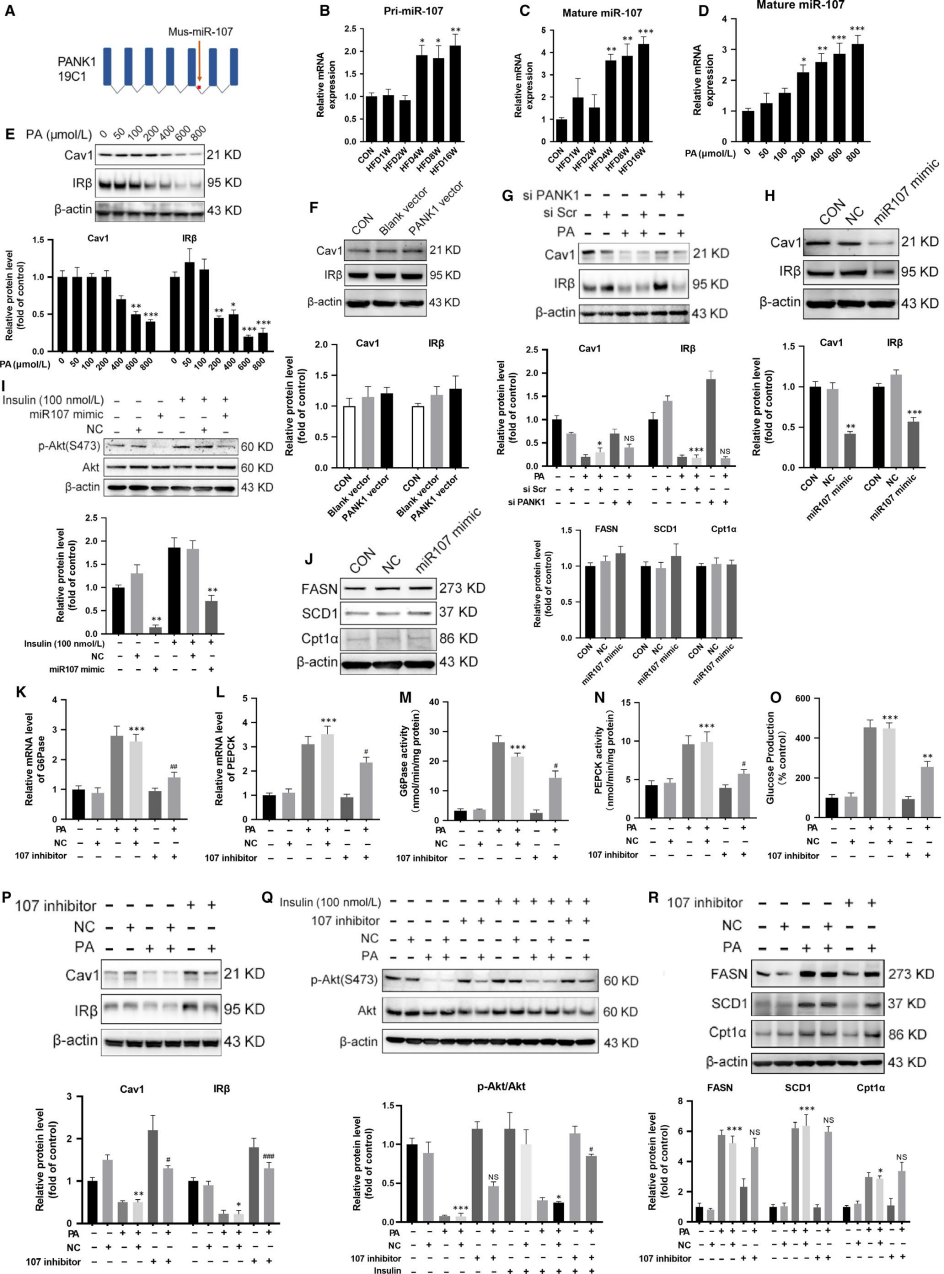
MiR‐107 within an intron of *PANK1* gene induced insulin resistance by targeting caveolin‐1. A, Genomic context of *PANK1* showed that miR‐107 located within intron of *PANK1* gene. *PANK1* gene are shown with exons as rectangles and introns as crooked lines. B and C, The mice were fed on HFD, and the levels of pri‐miR‐107 and mature miR‐107 in the liver tissue were measured at 1st, 2nd, 4th, 8th and 16th week of HFD feeding (n = 6). D, The AML12 cells were treated with 0, 50, 100, 200, 400, 600 and 800 μmol/L palmitate acid (PA) for 24 h, and the mature miR‐107 was measured by real‐time RT‐PCR. E: The protein levels of caveolin1 (Cav1) and IRβ were measured by Western blotting. F, The AML12 cells were transfected with blank vector or PANK1 vector. The protein levels of Cav1 and IRβ were measured by Western blotting. G, The AML12 cells were treated with scrambled small interfering RNA (si Scr) or PANK1 small interfering RNA (si PANK1). After 24 h, the cells were exposed to palmitate acid (600 nmol/L, PA) for 24 h. The protein levels of Cav1 and IRβ were measured by Western blotting. H: The AML12 cells were treated with negative control (NC) or miR‐107 mimic for 48 h. The protein levels of Cav1 and IRβ in AML12 cells were measured by Western blotting. I: Before harvest, cells were stimulated with 100 nmol/L insulin for 20 min and protein expressions of p‐Akt (S473) and Akt were measured. J, The protein levels of FASN, SCD1 and Cpt1α in AML12 cells were measured. K and L, The AML12 cells were treated with negative control (NC) or miR‐107 inhibitor. After 24 h, the cells were exposed to palmitate acid (600 nmol/L, PA) for 24 h. The mRNA levels of G6Pase and PEPCK in AML12 cells were measured. The activity of G6Pase and PEPCK in AML12 cells was measured (M and N). Glucose production measured in AML12 cells (O). The protein levels of Cav1and IRβ in AML12 cells were measured by Western blotting (P). Before harvest, cells were stimulated with 100 nmol/L insulin for 20 min and protein expressions of p‐Akt (S473) and Akt were measured (Q). R, The protein levels of FASN, SCD1 and Cpt1α in AML12 cells were measured. All values are presented as mean ± SEM. Data in B‐E, *P < .05, **P < .01, ***P < .001 (vs CON). Data in G, *P < .05, ***P < .001 (vs si Scr); NS (vs PA + si Scr). Data in H‐J, **P < .01, ***P < .001 (vs NC). Data in K‐R, *P < .05, **P < .01, ***P < .001 (vs NC); ^#^ *P* < .05, ^##^ *P* < .01, ^###^ *P* < .001 (vs PA + NC). n = 3 independent experiments

Genomic context of *PANK1* showed that miR‐107 located within intron of *PANK1* gene (Figure 4A). It was reported that PANK1 and miR‐107 shared the same transcriptional start site and were transcribed simultaneously.^10^ MiR‐107 has been reported to inhibit the translation of Cav1 and impair the stabilization of insulin receptor β (IRβ), resulting in IR.^8^ Our results showed that the expression of pri‐miR‐107 and mature miR‐107 in the liver tissue of mice was up‐regulated stimulated by HFD (Figure 4B,C). In AML12 cells, PA incubation increased the expression of miR‐107 whereas inhibited the expression of Cav1 and IRβ (Figure 4D,E). The inhibition of Cav1 and IRβ was PANK1‐independent as manipulation of PANK1 protein level had no influence on the expression of Cav1 and IRβ (Figure 4F,G). On the contrary, overexpression of miR‐107 with mimic was able to inhibit the protein expression of Cav1 and IRβ, and blunt insulin signalling indicated by the impaired phosphorylation of Akt stimulated by insulin (Figure 4H,I). However, overexpression of miR‐107 exerted no effect on the expression of enzymes involved in lipid metabolism (Figure 4J).

To further illustrate the role of miR‐107 in PA‐induced metabolic reprogram and IR, the AML12 cells were treated with miR‐107 inhibitor. The results showed that PA incubation significantly increased the mRNA expression and activity of enzymes modulating gluconeogenesis, which was partially reversed by inhibition of miR‐107 (Figure 4K‐N). Consistently, the glucose production induced by PA was significantly decreased by inhibition of miR‐107 (Figure 4O). In addition, inhibition of miR‐107 partially recovered IR induced by PA (Figure 4P,Q). However, inhibition of miR‐107 exerted no effect on the expression of enzymes involved in lipid metabolism (Figure 4R). The above data suggested that PANK1 and miR‐107 regulated metabolic reprogramming and insulin signalling, respectively.

### 3.4 Activation of P53 induced metabolic reprogramming through transcriptional activation of PANK1

**Figure 5:**
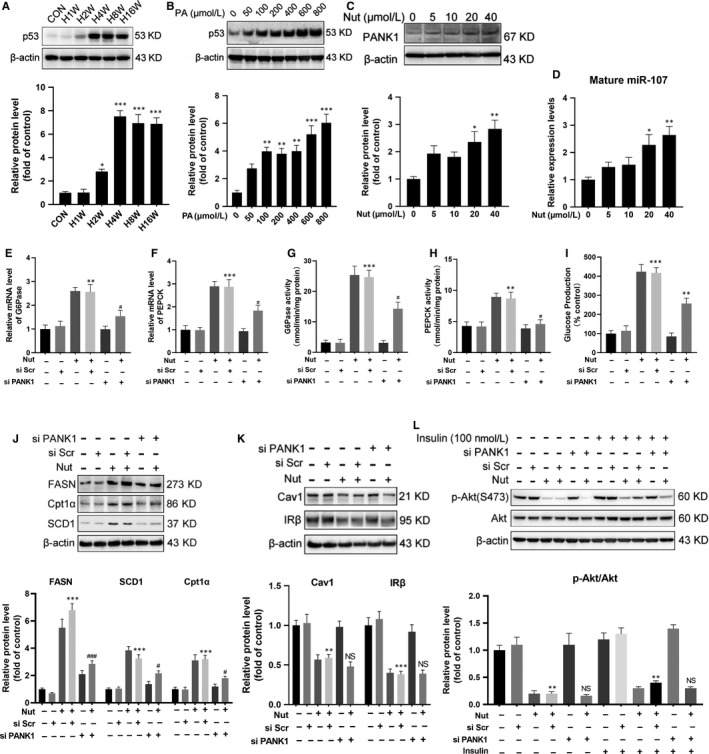
Activation of P53 induced metabolic reprogramming through transcriptional activation of *PANK1*. A, The mice were fed on HFD, and the protein level of P53 in the liver tissue was measured by Western blotting at 1st, 2nd, 4th, 8th and 16th week of HFD feeding (n = 6). B, The AML12 cells were treated with 0, 50, 100, 200, 400, 600 and 800 μmol/L palmitate acid (PA) for 24 h, and the protein level of P53 was measured by Western blotting. C: The AML12 cells were treated with 0, 5, 10, 20 and 40 μmol/L Nutlin‐3a (Nut) for 24 h, and the protein level of PANK1 was measured by Western blotting. D, The level of mature miR‐107 in AML12 cells was measured by real‐time RT‐PCR. E and F, The AML12 cells were treated with scrambled small interfering RNA (si Scr) or PANK1 small interfering RNA (si PANK1). After 24 h, the cells were treated with Nutlin‐3a (20 μmol/L, Nut) for 24 h. The mRNA levels of G6Pase and PEPCK in AML12 cells were measured. G and H, The activity of G6Pase and PEPCK in AML12 cells was measured. I: Glucose production measured in AML12 cells. J and K, The protein levels of FASN, SCD1, Cpt1α, Cav1 and IRβ were measured by Western blotting. L, Before harvest, cells were stimulated with 100 nmol/L insulin for 20 min and protein expressions of p‐Akt (S473) and Akt were measured. All values are presented as mean ± SEM. Data in A‐D, *P < .01, **P < .01, ***P < .001 (vs CON). Data in E‐L, **P < .01, ***P < .001 (vs si Scr); ^#^ *P* < .05, ^##^ *P* < .01, ^###^ *P* < .001 (vs Nut + si Scr). n = 3 independent experiments

P53 was shown to regulate carbohydrate, lipids and amino acid metabolism in addition to its critical role as a tumour suppressor.^19^ Our results showed that HFD significantly increased the expression of P53 in the liver, and PA incubation also increased the expression of P53 in AML12 cells (Figure 5A,B). P53‐binding site in the PANK1 promoter and the coregulation of PANK1 and its intronic miRNA‐107 were characterized.^10^ We confirmed that nutlin‐3a (Nut, P53 agonist) increased the expression of PANK1 and miR‐107 (Figure 5C,D). Nutlin‐3a incubation resulted in the enhancement of gluconeogenesis indicated as increased expression and activity of enzymes involved as well as enhanced glucose production, which was partially reversed by knocking down PANK1 (Figure 5E‐I). The enhancement of lipid metabolism induced by activation of P53 was almost abolished by siPANK1 treatment (Figure 5J). Furthermore, nutlin‐3a treatment suppressed the expression of Cav1 and IRβ as well as impaired insulin signalling, which was not reversed by knocking down PANK1 (Figure 5K‐L). These data showed that activation of P53 induced metabolic reprogramming and IR. The metabolic reprogramming induced by P53 was PANK1‐dependent, but the IR was PANK1‐independent.

### 3.5 Activation of P53 induced IR through transcriptional activation of miR‐107

**Figure 6:**
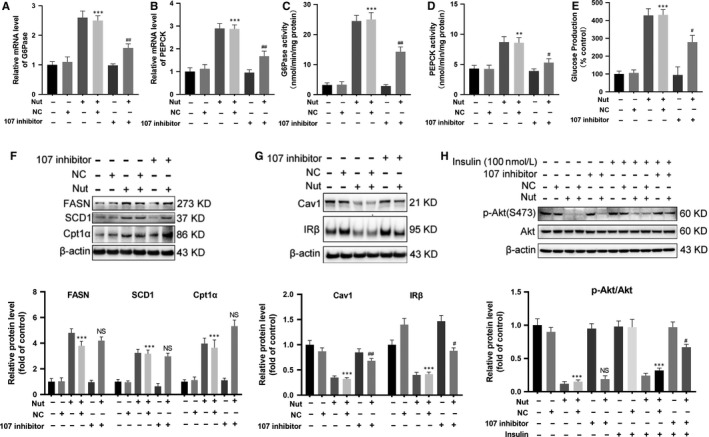
Activation of P53 induced insulin resistance through transcriptional activation of miR‐107. The AML12 cells were treated with negative control (NC) or miR‐107 inhibitor in the presence of Nutlin‐3a (20 μmol/L, Nut) for 24 h. A and B, The mRNA levels of G6Pase and PEPCK in AML12 cells were measured. C and D, The activity of G6Pase and PEPCK in AML12 cells was measured. E, Glucose production measured in AML12 cells. F and G, The protein levels of FASN, SCD1, Cpt1α, Cav1 and IRβ in AML12 cells were measured by Western blotting. H, Before harvest, cells were stimulated with 100 nmol/L insulin for 20 min and protein expressions of p‐Akt (S473) and Akt were measured. All values are presented as mean ± SEM. **P < .01, ***P < .001 (vs NC); ^#^ *P* < .05, ^##^ *P* < .01, ^##^ *P* < .01 (vs Nut + NC). n = 3 independent experiments

The enhanced gluconeogenesis induced by activation of P53 with nutlin‐3a was attenuated not only by knocking down PANK1 but also by incubation with miR‐107 inhibitor (Figure 6A‐E). However, we did not observe any influence of miR‐107 inhibition on the expression of enzymes involved in lipid metabolism (Figure 6F). Moreover, inhibition of miR‐107 recovered the expression of Cav1 and IRβ in AML12 cells upon nutlin‐3a treatment (Figure 6G). Also, inhibition of miR‐107 almost abolished the decreased phosphorylation of Akt induced by nutlin‐3a (Figure 6H), which implied that the ameliorating IR maybe contributed to the decreased gluconeogenesis as insulin suppresses hepatic output of glucose. These data showed that activation of P53 induced gluconeogenesis and IR through transcriptional activation of miR‐107.

### 3.6 Inhibition of P53 ameliorated PA‐induced metabolic reprogramming and IR

**Figure 7:**
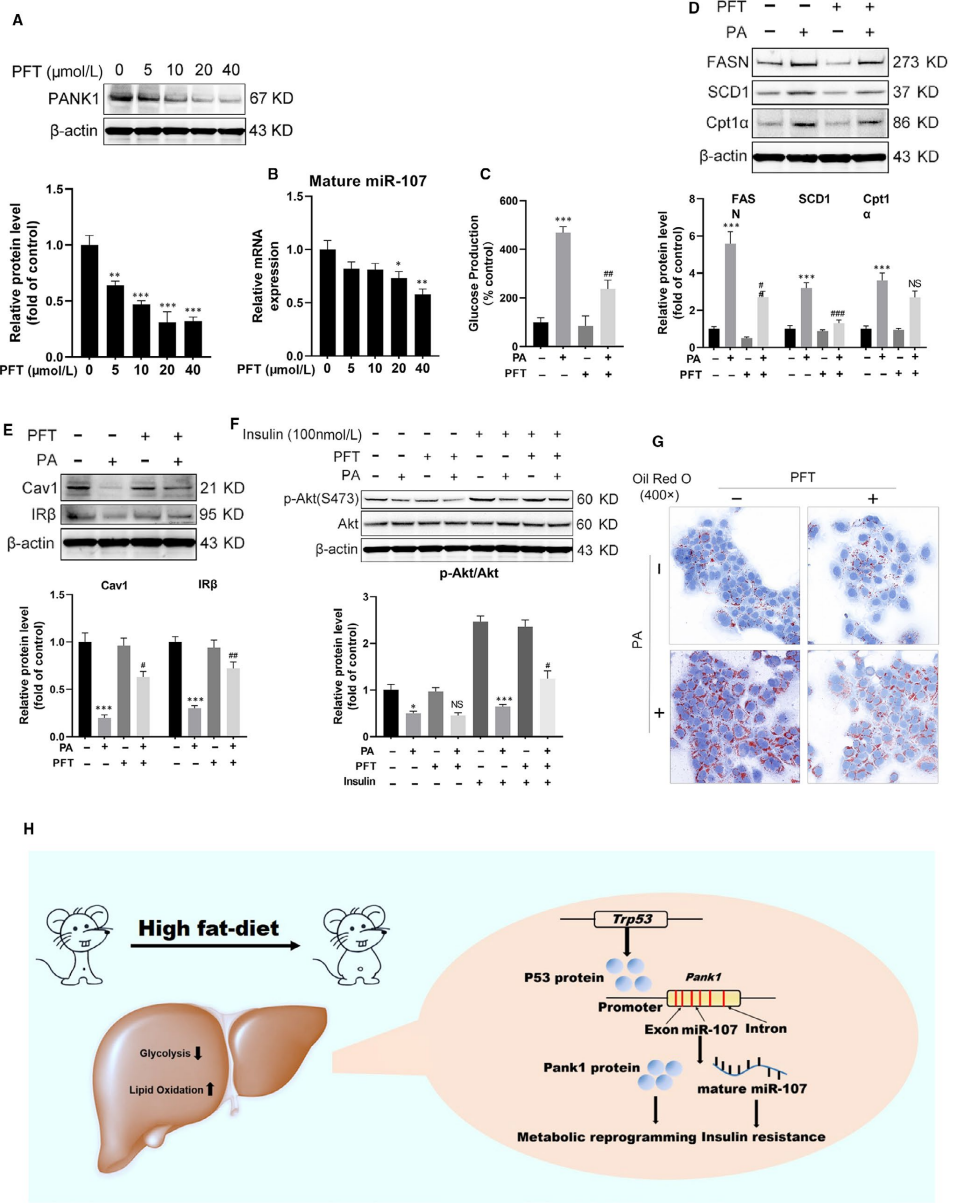
Inhibition of P53 ameliorated metabolic reprogramming and insulin resistance induced by palmitate acid. A, The AML12 cells were treated with 0, 5, 10, 20 and 40 μmol/L Pifithrin‐α hydrobromide (PFT) for 24 h, and the protein level of PANK1 was measured by Western blotting. B, The mature miR‐107 was measured by real‐time RT‐PCR. C, Glucose production measured in AML12 cells. D and E, The AML12 cells were treated with or without PFT (20 μmol/L) in the presence of palmitate acid (600 μmol/L) for 24 h. The protein levels of FASN, SCD1, Cpt1α, Cav1 and IRβ in AML12 cells were measured. F, Before harvest, cells were stimulated with 100 nmol/L insulin for 20 min and protein expressions of p‐Akt (S473) and Akt were measured. G, Representative images of Oil Red O staining are shown (400×). H, Proposed mechanisms by which high‐fat diet–induced metabolic reprogramming and insulin resistance through P53 activation of PANK1 and miR‐107, respectively. All values are presented as mean ± SEM. Data in A and B, *P < .05, **P < .01, ***P < .001 (vs CON). Data in C‐F, *P < .05, ***P < .001 (vs CON); ^#^ *P* < .05, ^##^ *P* < .01, ^###^ *P* < .001 (vs PA). n = 3 independent experiments

Then, we investigated whether inhibition of P53 reverses PA‐induced metabolic reprogramming and IR. The results showed that P53 antagonist pifithrin‐α hydrobromide (PFT) down‐regulated the expression of PANK1 and miR‐107 (Figure 7A,B). PFT incubation recovered glucose production (Figure 7C) and abolished the enhanced lipid metabolism induced by PA incubation as indicated by the decreased expression of FASN, SCD1 and Cpt1α (Figure 7D). Moreover, PFT was shown to recover the expression of Cav1 and IRβ as well as ameliorate the phosphorylation of Akt in AML12 cells upon PA treatment (Figure 7E,F). In addition, PFT incubation alleviated the lipid droplet formation in AML12 cells induced by PA, which was determined with oil red O staining (Figure 7G). These data demonstrated that inhibition of P53 ameliorated PA‐induced metabolic reprogramming and IR. Proposed mechanisms by which high‐fat diet–induced metabolic reprogramming and insulin resistance through P53 activation of PANK1 and miR‐107, respectively (Figure 7H).

## 4 DISCUSSION

Multiple research intends to illuminate the underlying molecular mechanism through which HFD induced metabolic syndrome and diabetes. Our data confirmed that HFD led to IR and metabolic abnormalities which, at least, in part was dependent on a new molecular pathway P53/PANK1/miR107. The balance between glucose and lipid utilization for energy supply was disrupted by HFD, and the imbalance was a pivotal cause for the development of IR.

High‐fat diet is a widely adopted method to induce obesity, non‐alcoholic fatty liver and T2DM, in which IR is widely considered the ‘common soil’ of those metabolic disorders.^20^, ^21^, ^22^ Maternal obesity is afflicted with many maternal obstetric complications in the offspring including high blood pressure, obesity, gestational diabetes and increased perinatal morbidity.^23^ Several factors are involved in the progression of IR induced by HFD including inflammation, excessive ROS, abnormal immune response, lipotoxicity and altered glycosylation.^1^, ^24^, ^25^, ^26^, ^27^, ^28^ Recently, an important role for protein quality control, particularly autophagy, has been depicted in the maintenance of metabolic homeostasis.^29^ However, the mechanism underlying IR is far from clarified. Our data showed that the lipogenesis, lipid accumulation, lipid oxidation and gluconeogenesis in liver was inhanced induced by HFD, while the glycolysis was suppressed. Because of HFD, the glycolytic enzymes GCK and PFK‐1 in the liver decreased, whereas the expression of G6Pase and PEPCK involved in gluconeogenesis increased. Moreover, the expression of enzyme (Cpt1α) regulating oxidation of fatty acids and enzymes (FASN, SCD1) involved in lipid synthesis increased. However, the alteration in glucose and lipid metabolism induced by HFD in the liver is controversial^18^ and this discrepancy might attribute to the variance in the components of HFD, the duration of HFD, the tissue samples, animal species and even the experimental protocols. In the present study, we demonstrated that long‐term HFD shifted the fuel preference from glucose towards fatty acids and this metabolic reprogramming led to IR directly.

CoA is consumed massively in the process of FAO and synthesis. Thus, the biosynthesis of CoA needs to be enhanced during HFD. Pantothenate acid is the precursor for biosynthesis of CoA, which is the predominant acyl group carrier in cells and participates in over 100 intermediary metabolic reactions.^6^ Pantothenate is phosphorylated to yield 4‐phosphopantothenate catalysed by PANK and PANK activity controls the cellular level of CoA in mammals. The PANK activity in the liver varied in conjunction with CoA levels, and increased in diabetes or treatment with hypolipidemic agents.^30^ Reduction in hepatic CoA because of PANK1 deficiency or chemical inhibition of PANK isoforms results in reduced FAO and hypoglycaemia, demonstrating that modulation of CoA has a direct impact on glucose production.^31^ In particular, *PANK1*‐deficient mice are unable to convert pyruvate, oxaloacetate or glycerol into glucose.^4^ Our results showed that HFD increased the expression of PANK1 in the liver. Furthermore, overexpression of PANK1 enhanced the metabolism of lipid metabolism as well as the gluconeogenesis in AML12 cells.

Enhanced FAO during HFD is an adaptation to excessive fatty acids supply, which maintains the energy generation but contributes to the development of IR simultaneously. Our data in AML12 cells indicated that IR induced by PA was independent to the expression of PANK1, which is consistent with the previous data in vivo.^4^ Genomic context of *PANK1* showed that miR‐107 located within intron of *PANK1* gene and was co‐transcribed as reported in tumour cells and validated in AML12 cells.^10^ MiR‐107 was reported to inhibit the translation of Cav1, which participated in the formation of caveolae and assisted IRβ anchoring on the plasma membrane.^8^ Our results showed that PA induced IR and inhibited the expression of Cav1 and IRβ in a dose‐dependent manner. Furthermore, inhibition of miR‐107 ameliorated IR but exerted no influence on FAO upon PA treatment. Metabolic reprogramming and IR induced by HFD or PA was PANK1 protein and miR‐107 mediated, respectively, although PANK1 and miR‐107 were transcribed from the same gene *PANK1*. Interestingly, inhibition of miR‐107 significantly decreased gluconeogenesis upon PA treatment. This may be attribute to the improved insulin sensitivity.

Many studies have focused on the role of p53 in metabolism and IR.^32^ Several studies have shown that P53 is induced by HFD in various tissues. Zoltan Derdak demonstrated that pharmacologic inhibition of p53 with pifithrin‐α p‐nitro attenuates hepatic steatosis and liver injury in C57BL/6 mice fed HFD for 8 weeks.^11^ Moreover, systemic pifithrin‐α administration considerably diminished P53 levels and ameliorated Akt phosphorylation in all peripheral tissues including adipose tissues and alleviated IR.^33^ As Mdm2 protein functions as an E3 ubiquitin ligase that inhibits p53 transcriptional activation, the *Mdm2^C305F^* mice are resistant to HFD‐induced obesity and obesity‐associated illnesses.^34^ Our results showed that P53 protein levels were markedly elevated in the liver tissue induced by HFD for 12 weeks and PA incubation enhanced the expression of P53 in AML12 cells. Mechanically, p53 has been shown to regulate glycolysis and mitochondrial respiration through transcriptional control of several metabolic genes.^35^ It was reported that P53‐mediated PTEN overexpression might be involved in impaired insulin signalling through inducing senescence‐like phenotypes and producing inflammatory cytokines.^36^ P53 also repressed the expression of glucose transporters GLUT1/3/4, thereby decreasing cellular uptake of glucose and subsequent glycolysis.^37^ We showed that P53 activation enhanced the FAO and gluconeogenesis, but suppressed glycolysis, which was like the alteration induced by HFD. However, pifithrin‐α hydrobromide significantly recovered the lipid metabolism and gluconeogenesis induced by PA in AML12 cells. Moreover, pifithrin‐α hydrobromide recovered the expression of Cav1 and IRβ as well as increased the phosphorylation of Akt upon PA treatment. Furthermore, P53 regulated PANK1 expression and activity directly.^7^ Our data showed that nutlin‐3a increased the expression of PANK1 and miR‐107. P53 activation induced metabolic reprogramming and IR, which was mediated by PANK1 and miR‐107, respectively.

The metabolism of nutrients is one of the fundamental features of life during which the ingredients of organism are renewed and energy in the form of ATP is synthesized. Metabolism is elaborately modulated through regulating the expression and activity of key enzymes catalysing the conversion of substrates. Metabolic pathway is programed and could be reprogrammed under the physiological or pathological changes. Metabolic reprogramming was first introduced in tumour tissues and was considered as the adaptation of cancer cells to local environment such as hypoxia. It seemed reasonable that the balance of glucose and lipids metabolism rebuild by HFD was regarded as physiological adaptation. It is not the liver that decides what kind of substrates to utilize and adaptation is the dominator. However, when the liver enhanced the ability to catalyse more lipids and up‐regulated the expression of PANK1, miR‐107 was up‐regulated simultaneously, which induced the progression of IR. It was controversial to classify IR to the side effect of metabolic programming as IR accompanied by decreased glycolysis and increased gluconeogenesis could be considered as a part of metabolic adaption. However, it is undeniable that IR is the prelude to diabetes. Maybe metabolic reprogramming induced by HFD is another example that physiological adaptation beyond limitation is the initiation of pathological changes.

There are still some limitations in this study. Firstly, whether the mechanism still works in vivo require experimental verification. Secondly, because of lack of radioactive experimental conditions, the present study failed to detect the level of CoA and the activity of PANK1. Lastly, long‐term HFD increases the incidence rate of carcinogenesis and the present study showed that P53 was up‐regulated by HFD, which seems to be incompatible. It seems that the role of P53 in carcinogenesis and metabolic syndrome is complicated and need to be further explored.

Conclusively, our study has demonstrated that P53/PANK1/miR‐107 is the link between metabolic reprogramming and IR induced by HFD. In addition, the results distinguished the different roles of PANK1 and its intron miR‐107 in metabolic regulation, which will provide more accurate intervention targets for the treatment of metabolic diseases.

## CONFLICT OF INTEREST

No potential conflicts of interest relevant to this article were reported.

## AUTHOR CONTRIBUTIONS

LY and LC designed research and overall study, supervised the experiments, analysed the results, and wrote the article. LC and BZ performed the real‐time RT‐PCR and Western blotting experiments. ZH.L. and SM.Z. cultured the cells. JL and XM.G. measured the glucose production. MJ, HT.G. and XJ.W. participated in the in vivo study, performed the IPGTT and IPITT experiments. ZH.L. analysed data and contributed to the discussion. NF and RF analysed data. JM.P. and MJ.X. reviewed/edited the manuscript. LC and LY are the guarantors of this work and, as such, had full access to all the data in the study and take responsibility for the integrity of the data and the accuracy of the data analysis.

## Data Availability

The data used to support the findings of this study are available from the corresponding author upon request.
