# Supplementary figures and images for: P53/PANK1/miR‐107 signalling pathway spans the gap between metabolic reprogramming and insulin resistance induced by high‐fat diet
Source: J Cell Mol Med. 2020 Feb 12;24(6):3611–24. doi: 10.1111/jcmm.15053 (PMC7131928; doi:10.1111/jcmm.15053)

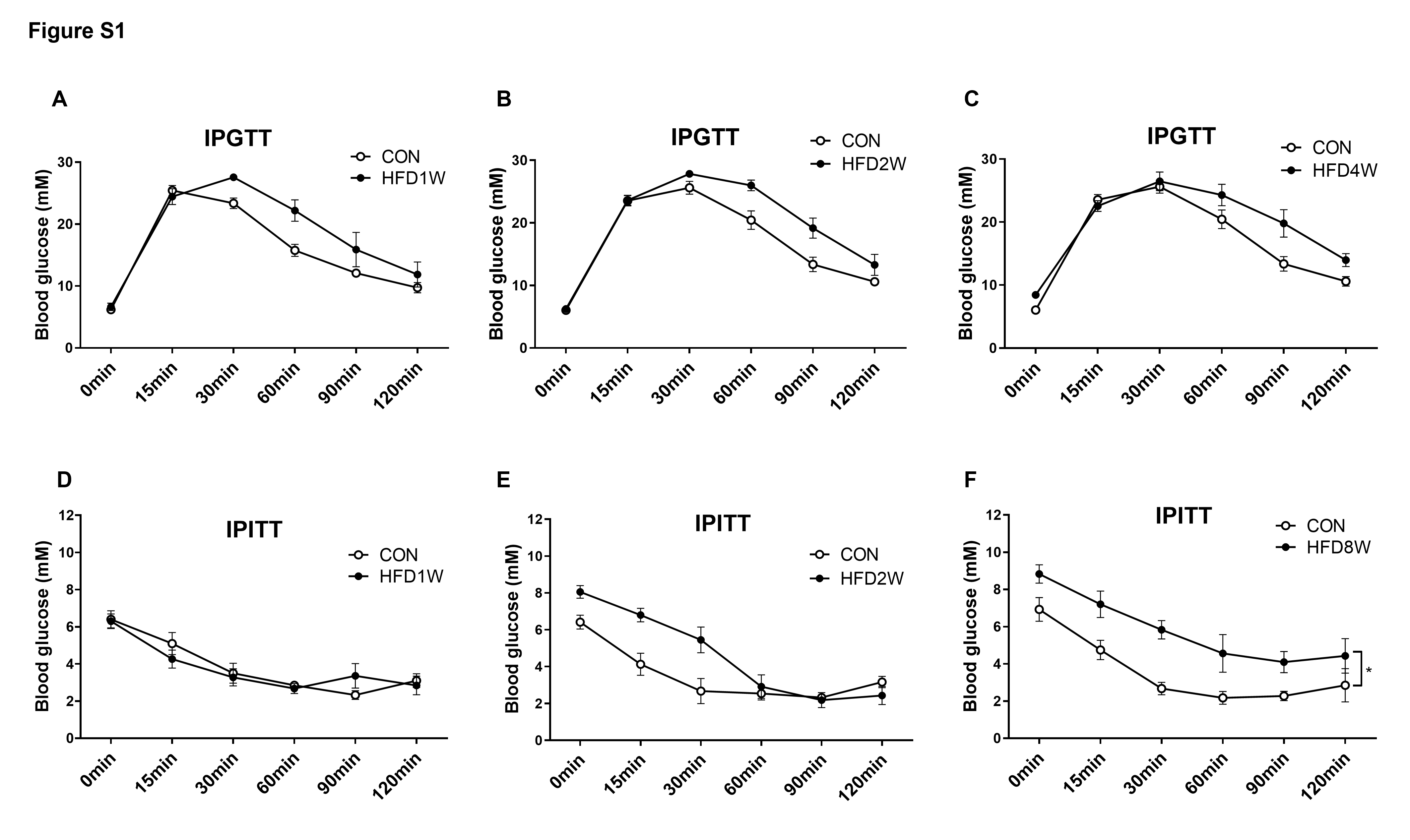

Supplement: Supplementary file 1 [file JCMM-24-3611-s001.tif]

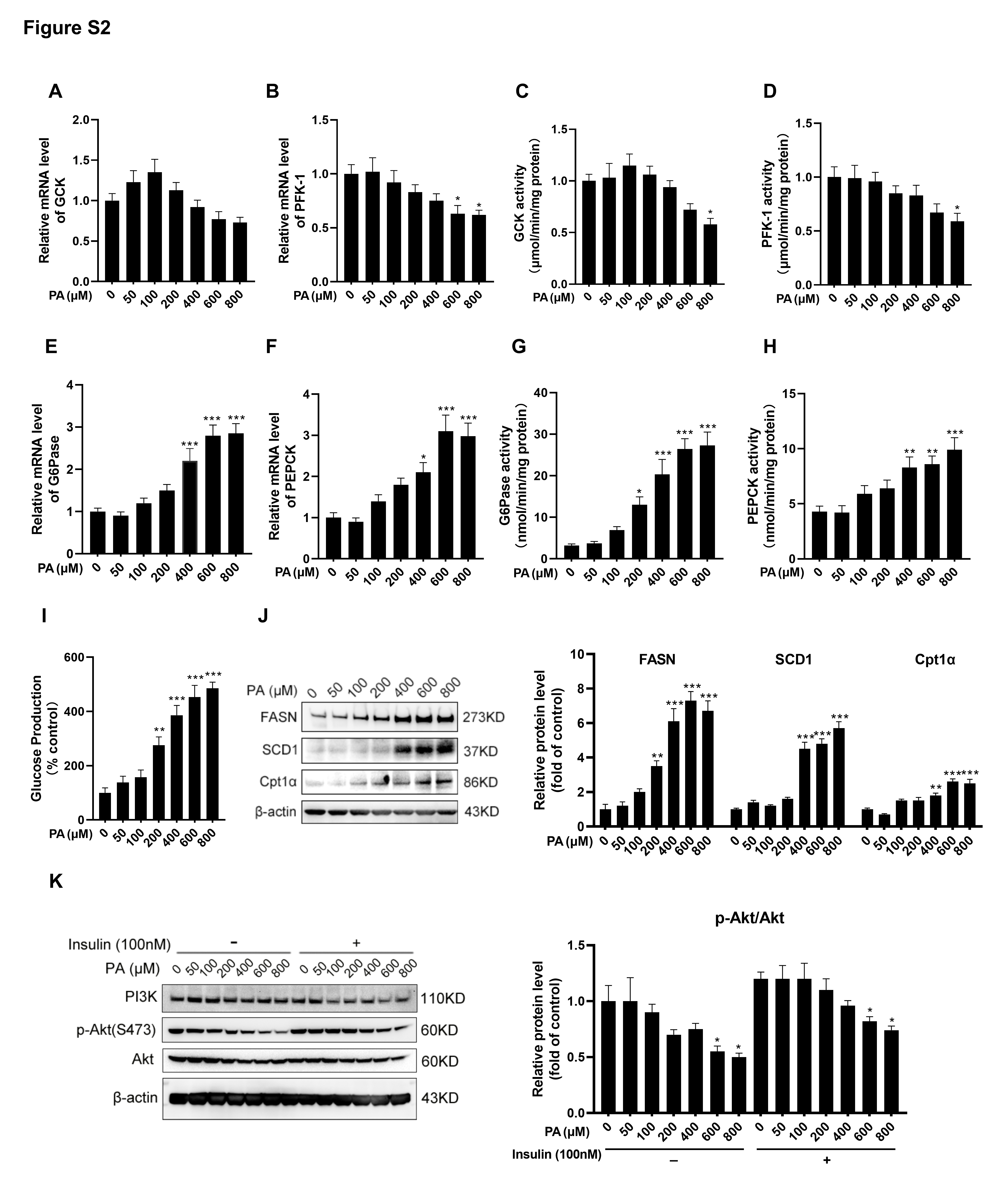

Supplement: Supplementary file 2 [file JCMM-24-3611-s002.tif]
