# Supplementary material for: P53/PANK1/miR‐107 signalling pathway spans the gap between metabolic reprogramming and insulin resistance induced by high‐fat diet
Source: J Cell Mol Med. 2020 Feb 12;24(6):3611–24. doi: 10.1111/jcmm.15053 (PMC7131928; doi:10.1111/jcmm.15053)
Supplement: Supplementary file 3 [file JCMM-24-3611-s003.docx]

|  | **Target gene** | **FORWARD** | **REVERSE** |
| --- | --- | --- | --- |
| 1 | Gck | CCTGCTACTATGAAGACCGCCAATG | GCCTTCCACCAGCTCCACATTC |
| 2 | PFK-1 | CGCATCAAGCAGTCAGCCTCAG | AGCCAGGTAGCCACAGTAGCC |
| 3 | G6pc | TTGTGTCTGTGATTGCTGACCTGAG | GCTGTTGCTGTAGTAGTCGGTGTC |
| 4 | Pepck1 | GCAGTGAGGAAGTTCGTGGAAGG | GTCAGTGAGAGCCAGCCAACAG |
| 5 | Fasn | CCGTGTGACCGCCATCTATATCG | CGTGAGGTTGCTGTCGTCTGTAG |
| 6 | SCD1 | CTCATGGTCCTGCTGCACTTGG | TGTGGCTCCAGAGGCGATGAG |
| 7 | Cpt1a | GAATCTGGATGGCTATGGTCAAGGTC | AGTGCTGTCATGCGTTGGAAGTC |
| 8 | miR-107 | CCGAGCTTCTTTACAGTGTTGCCTTG |  |
| 9 | Pri-miR-107 | TGTGCTTTCAGCTTCTTTACAGTG | GCTCTCTGTGCTTTGATAGCC |

**Table S1. Primers used for real-time PCR.** This table describes the primers used for evaluation of mRNA expression in liver and AML12 cells.

**Figure S1. Systemic insulin sensitivity was evaluated in mice on HFD**. Eight-week-old male C57BL/6 mice were randomized into two groups: standard normal diet (CON) and high-fat diet (HFD). A-C: Intraperitoneal glucose tolerance test (IPGTT) were performed in mice on HFD for 1 week, 2 weeks 4 weeks (n = 6). D-F: Intraperitoneal insulin tolerance test (IPITT) were performed in mice on HFD for 1 week, 2 weeks 8 weeks (n = 6). All values are presented as mean ± SEM. ^*^*P*<0.05 (*vs*. CON).

**Figure S2. Palmitate acid induces metabolic reprogramming and insulin resistance in cultured hepatocytes.** AML12 cells were treated with 0 μM, 50 μM, 100 μM, 200 μM, 400 μM, 600 μM and 800 μM palmitate acid (PA) for 24 h. A-D: The mRNA level and activity of GCK and PFK-1 in AML12 cells were measured. E-H: The mRNA level and activity of G6Pase and PEPCK in AML12 cells were measured. I: Glucose production measured in AML12 cells. J: The protein levels of FASN, SCD1 and Cpt1α in AML12 cells were measured by western blotting. K: Before harvest, cells were stimulated with 100 nM insulin for 20 min and protein expressions of PI3K, p-Akt (S473) and Akt were measured by western blotting. All values are presented as mean ± SEM. ^*^*P*<0.05, ^**^*P*<0.01, ^***^*P*<0.001 (*vs*. CON). n = 3 independent experiments.
